# The Role of Reactive Oxygen Species (ROS) in the Formation of Extracellular Traps (ETs) in Humans

**DOI:** 10.3390/biom5020702

**Published:** 2015-05-04

**Authors:** Walter Stoiber, Astrid Obermayer, Peter Steinbacher, Wolf-Dietrich Krautgartner

**Affiliations:** Biomedical Ultrastructure Research Group, Department of Cell Biology, University of Salzburg, Hellbrunnerstrasse 34, Salzburg A-5020, Austria; E-Mails: astrid.obermayer@sbg.ac.at (A.O.); peter.steinbacher@sbg.ac.at (P.S.); wolf.krautgartner@sbg.ac.at (W.-D.K.)

**Keywords:** neutrophils, NETs, autophagy, NADPH oxidase, MAPK/ERK, inflammation

## Abstract

Extracellular traps (ETs) are reticulate structures of extracellular DNA associated with antimicrobial molecules. Their formation by phagocytes (mainly by neutrophils: NETs) has been identified as an essential element of vertebrate innate immune defense. However, as ETs are also toxic to host cells and potent triggers of autoimmunity, their role between pathogen defense and human pathogenesis is ambiguous, and they contribute to a variety of acute and chronic inflammatory diseases. Since the discovery of ET formation (ETosis) a decade ago, evidence has accumulated that most reaction cascades leading to ET release involve ROS. An important new facet was added when it became apparent that ETosis might be directly linked to, or be a variant of, the autophagy cell death pathway. The present review analyzes the evidence to date on the interplay between ROS, autophagy and ETosis, and highlights and discusses several further aspects of the ROS-ET relationship that are incompletely understood. These aspects include the role of NADPH oxidase-derived ROS, the molecular requirements of NADPH oxidase-dependent ETosis, the roles of NADPH oxidase subtypes, extracellular ROS and of ROS from sources other than NADPH oxidase, and the present evidence for ROS-independent ETosis. We conclude that ROS interact with ETosis in a multidimensional manner, with influence on whether ETosis shows beneficial or detrimental effects.

## 1. Introduction and Background

**Figure 1:**
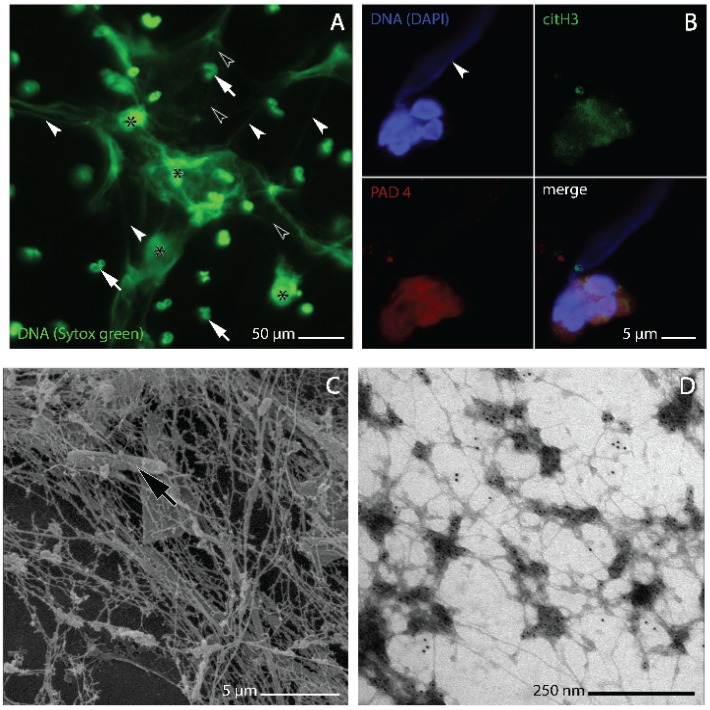
Morphology of NETs and NET-forming human neutrophils as analyzed by confocal laser microscopy (**A**, **B**), and scanning and transmission electron microscopy (**C** and **D**, respectively). (**A**) NETs generated *in vitro* from human neutrophils isolated from whole venous blood using a standard gradient separation medium containing sodium metrizoate and Dextran 500 [34]. NETosis induction by stimulation with 1 µM fMLP followed the procedure described by [35]. DNA was stained with sytox^®^ green. At light microscopic resolution, NETs appear as irregular cloudy structures in which dense clusters of brightly stained extracellular DNA (asterisks) merge with more faintly stained dilated areas in which the DNA is more thinly spread and forms a meshwork of threads (white arrowheads). Lobulated nuclei of non-NET forming neutrophils (white arrows) are found within the meshwork as well as outside of it, some being slightly out of focus; (**B**) elongated plume of NET-DNA (arrowhead) protruding from one of two attached NET-forming neutrophils from the sputum of a patient with chronic obstructive pulmonary disease (COPD). The cells are immunostained for peptidyl arginine deiminase 4 (PAD4, red) and citrullinated histone 3 (citH3, green), DNA is stained with 4',6-diamidino-2-phenylindole (DAPI, blue). Overlapping PAD4 and citH3 staining at nuclear and cytoplasmic sites is characteristic of NET-forming neutrophils (*cf.* [9,36]) and conforms with the observation of [37] that histone H3 deimination by PAD4 is not entirely confined to the nucleus; (**C**) bacterium (arrow) entangled in NETs from the sputum of a COPD patient; (**D**) on-grid preparation of *in vitro* generated NETs (procedures as described for A above) immunogold stained for the enzyme neutrophil elastase, one of the key protein components of NETs.

Extracellular traps (ETs) are reticulate formations of extracellular DNA associated with antimicrobial molecules (Figure 1A–D). Their formation, mainly by neutrophils and other cells of the immune system (eosinophils, macrophages, mast cells) in a distinctive process of cell death, has been identified as an important evolutionarily conserved mechanism of vertebrate innate immune defense [1,2,3,4]. First described in 2004 in humans [5], the ability of cells to release ETs is now known to occur not only in mammals but is also found in immune cells of birds and fish (e.g., [6,7,8]). ETs constitute complex three-dimensional web-like scaffolds of DNA strands with dimensions down to 2 nm, the size of individual double helices (Figure 1D) [9]. These scaffolds are decorated with histones and other molecules, including elastase (Figure 1B,D), myeloperoxidase (MPO), bactericidal permeability-increasing protein (BPI), cathepsin G and other proteinases that are all antimicrobially effective [5,9,10,11,12,13,14]. ETs have been shown to aid the entrapment and/or removal of bacterial, fungal, protist and even platyhelminth pathogens (e.g., [15,16,17,18,19]). They are also formed during viral infections, probably exerting a cell protective role [20,21]. However, the protein components of ETs have also been identified as toxic to host cells (e.g., [20,22]) and as potent triggers of autoimmunity [23,24]. Thus, since their discovery [5], ETs have been established in an ambiguous role between pathogen defense and host tissue damage [25].

The generation and release of ETs, specifically by neutrophils, has been shown to be induced by a variety of internal and/or pathogen derived molecular signals. These signals include chemokines with CXC motif such as interleukin 8 (IL-8) [5,26], but also tumor necrosis factor alpha (TNF α), lipopolysaccharides (LPS), formylated peptides such as *N*-formyl-methionyl-leucyl-phenylalanine (fMLP) (Figure 1A and D), placental syncytiotrophoblast microparticles, antineutrophil cytoplasmic antibodies (ANCAs), and pharmacological agents, such as phorbol myristate acetate (PMA) [2,5,23,24,27]. According to recent evidence, antibody (IgA) binding by phagocyte FcαRI-receptors [28] and binding of neutrophil FcγRIIIb receptors to immune complexes immobilized in the extracellular matrix [29] are also likely to play an inductive role.

Mechanisms of ET formation (ETosis) have been found to vary in relation to the signaling pathways involved and in the morphological execution of the process, enabling more than one mechanism per cell type. This is well documented for PMA and fMLP induced pathways, and for the polymorphisms of ET formation in neutrophils (see below under Section 2, Section 3, Section 4, Section 5, Section 6, Section 7 and Section 8). There is also evidence that some types of ETosis leave the cells viable [30,31], a subject dealt with in more detail in Section 7 below.

Despite this heterogeneity, ETosis is mainly a distinctive process of cell death involving full chromatin decondensation and break-up of the cell membrane. This holds, primarily, for ET formation by neutrophils (NETosis), which is perhaps the best-investigated form of ETosis. Morphological research has identified a standard pattern referred to as the NETotic cascade. The steps of this cascade are well defined in the recent literature (e.g., [2,32,33]) and describe the progressive change from the undisturbed globular cell, via cytoplasmic and nuclear swelling, vacuolization, membrane protrusion, enzyme binding to DNA, histone citrullination and chromatin decondensation, to terminal membrane rupture and NET release (Figure 1B).

Independent of the mechanisms and cell types from which ETs derive, there is mounting evidence that they strongly contribute to severe acute illness and chronic inflammation when formed in excess or are insufficiently cleared (e.g., [25]). This has led to a surge of research activity into the details of these pathogenic effects, specifically, again, with regard to neutrophil generated ETs (NETs), which appear to be the most abundant.

## 2. ET Formation Is Linked to Reactive Oxygen Species (ROS) and Autophagy

ROS are a heterogeneous group of oxygen-containing molecules with high chemical reactivity, some being rendered unstable and extremely reactive due to an unpaired electron. This group includes peroxides, hypochlorous acid, hydroxyl radicals, singlet oxygen, and the superoxide anion, among other compounds. Physiological generation of ROS occurs either as byproducts of (redox) reactions in various cell organelles including mitochondria, peroxisomes, and endoplasmic reticulum, or by primary enzyme function, such as with oxidases and oxygenases. Such enzymes have long been associated with the respiratory burst of phagocytes (see below), but are now known to occur in virtually every type of cell and tissue [38,39,40].

**Figure 2:**
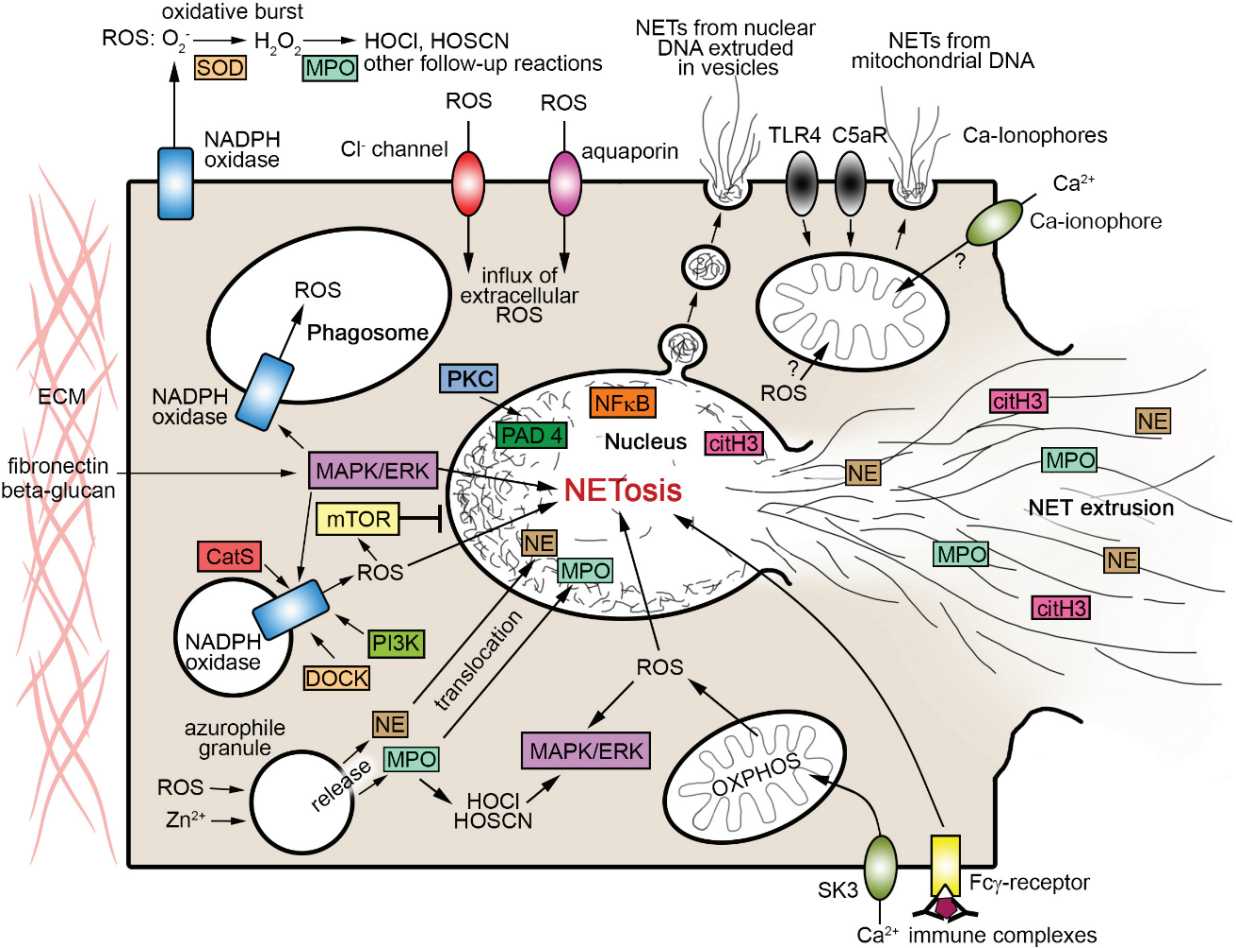
Scheme summarizing pathways of interaction between ROS and NET formation as addressed in the text. Arrows indicate directions of effects. Note that only some of the processes shown can co-occur. CatS cathepsin S, citH3 citrullinated histone H3, C5aR complement component 5a receptor, DOCK dedicator of cytokinesis proteins, ERK extracellular signal-regulated kinases, HOCl hypochlorous acid, HOCSN hypothiocyanous acid, MASPK mitogen-activated protein kinases, MPO myeloperoxidase, mTOR mammalian target of rapamycin, NE neutrophil elastase, NFκB nuclear factor kappa-light-chain-enhancer of activated B cells, OXPHOS oxidative phosphorylation, PAD4 peptidylarginine deiminase 4, PI3K phosphoinositide-3-kinase, PKC protein kinase C, SK3 small conductance calcium-activated potassium channel 3, SOD superoxide dismutase, TLR4 toll-like receptor 4.

An important finding in recent ET research is that ROS are apparently an integral part of most reaction cascades entailing the release of ETs [3,41,42] (Figure 2). As with the morphological pattern above, the evidence is mainly derived from ET formation by neutrophils (NETosis). Analysis from various perspectives has shown that NETosis involves ROS formation by the multienzyme complex NADPH oxidase (e.g., [1,3,18,28,32,42]) (see below under Section 3). Recent research provides strong evidence that NETosis is directly linked to (or is a variant of) the autophagy pathway [43,44]. Autophagy is a conserved process of lysosome-mediated intracelluar degradation [45] enabling the routine turnover of proteins and organelles. It further contributes to a wide spectrum of physiological functions including stress response, nutritional starvation management, tumor development and pathogen clearance (e.g., [46,47,48,49,50]). Protein kinase B (PKB/AKT), mammalian/mechanistic target of rapamycin (mTOR), and mitogen-activated protein kinases (MAPK, also known as extracellular signal-regulated kinases, ERK) have long been known to be important regulators of autophagy, mainly in work from tumor biology. Specifically, the phosphatidylinositol 3-kinase (PI3K)/AKT/mTOR signaling pathway is a negative regulator of both autophagy and apoptosis (e.g., [49,51,52,53]), while MAPK/ERK pathways act as positive autophagy regulators (e.g., [49,54,55]).

It is now known that the regulation of autophagy, and especially its association with ETosis, is closely tied to ROS in a multifactorial manner (e.g., [56,57]). Importantly, the level of intracellular ROS determines whether the autophagy reaction ends in NETosis [1,44,58,59]. Placed in a wider perspective, this association with NETosis strengthens the view that autophagy is not a “simple” mechanism of cell death alternative to apoptosis, but serves primarily to protect vertebrate cells from various types of harm including that from microbes (*cf.* [60]). However, the exact ways in which ROS interfere with the signaling network behind autophagy to initiate and/or promote NETosis are still incompletely understood, particularly in relation to how they contribute to the remodeling of the cell interior.

## 3. The Role of NADPH Oxidase-Derived ROS in NET Formation

NADPH (nicotinamide adenine dinucleotide phosphate) oxidases are a family of membrane bound multiprotein enzymes that generate ROS delivered into either extracellular or intracellular compartments. Phagocytes have long been known to express large amounts of NADPH oxidase residing in the plasma membrane and in phagosome membranes (Figure 2), but intermediate or low amounts of the enzyme occur in most—if not in all—mammalian cell types and tissues [40,61,62]. The phagocyte NADPH oxidase consists of a b-type cytochrome-containing transmembrane protein termed gp91^phox^ (also known as NOX2, see below), with four further “phox” (phagocytic oxidase) elements (p22^phox^, p40^phox^, p47^phox^, p67^phox^) and a guanosine triphosphatase (GTPase), usually belonging to the Ras-related C3 botulinum toxin substrate types Rac1 or Rac2 within the Rho (Ras homologue) family and Ras (rat sarcoma) superfamily of small GTPases. The gp91^phox^ subunit was found to have several homologues in nonphagocytic NADPH oxidases (NOX). Together with gp91^phox^, these homologues have been pooled in the NOX family, comprising NOX1, NOX2 (= gp91^phox^), NOX3, NOX4, NOX5, and also DUOX (dual oxidase) 1 and 2. Nonphagocytic NADPH oxidases differ from the phagocytic enzyme in molecular structure, subcellular location and biochemical function [40,62,63,64].

NADPH oxidases exist in different states of activation (resting, primed, active, or inactive). Signal molecules able to induce subunit assembly and activation include proinflammatory cytokines, lipopolysaccharides, Toll-like receptor (TLR) agonists, and chemical agents such as PMA [65], and are largely the same as those known to induce NETosis. Forming the functional enzyme requires the phosphorylation of protein subunits (e.g., p40^phox^) by MAPK, and the translocation of cytosolic components to membranes [65,66,67,68]. The situation is complicated by the fact that the regulatory pathways of NADPH oxidase assembly vary depending upon the molecular triggers. In neutrophils, this is exemplified by the difference between fMLP and PMA, the former acting via receptor-mediated pathways with downstream protein kinase involvement (e.g., [69,70], see also information on PI3K/AKT/mTOR pathways below) while the latter operates directly via protein-kinase C (e.g., [71,72]).

Once assembled and activated, NADPH oxidases transfer electrons from cytoplasmic NADPH across biological membranes and couple them to molecular oxygen, thus generating the superoxide radical anion ^●^O_2_^−^. This enables a cascade of ROS generation that continues with the rapid conversion of ^●^O_2_^−^·to hydrogen peroxide H_2_O_2_, either spontaneously or catalysed by superoxide dismutase (SOD) [40]. The process may then proceed to the MPO catalysed formation of hypochlorous acid (HOCl) from H_2_O_2_[73,74,75]. HOCl is able, in turn, to re-react with H_2_O_2_ to generate singlet molecular oxygen (^1^O_2_) and peroxyl radicals [76]. Availability of transition metals (specifically iron) may enable the H_2_O_2_ to undergo the Fenton reaction rendering the highly reactive hydroxyl radical (^●^OH) [39,40]. Further reactions may follow under given conditions, such as the production of nitric oxide (NO) by inducible nitric oxide synthase (iNOS), and may again entail consecutive reactions, e.g., with ^●^O_2_^−^ to form peroxynitrite [66,77].

It has been recognized for some time that neutrophils and other phagocytes produce large amounts of extracellular ROS (e.g., H_2_O_2_ and superoxide anion ^●^O_2_^−^) upon stimulation with a wide variety of agents. Due to a transient rise in oxygen demand, this behavior was originally named respiratory (or oxidative) burst (e.g., [40,78]). Plasma membrane-bound phagocyte NADPH oxidase is commonly thought to be the main source of ROS delivery to the extracellular space during respiratory bursts, and into engulfed phagosomes for microbial killing [65,79,80]. In addition, NADPH oxidase derived ^●^O_2_^−^ has been shown to promote microbial killing in the phagosome also indirectly by enabling the activation of serin proteases (e.g., cathepsin G and neutrophil elastase, NE) in neutrophil azurophilic granules via modulation of ion influx and pH [14,81,82] (there is, however, evidence suggesting that the importance of this mechanism for the microbicidal capacity of the neutrophils is limited [83]).

The circumstances of how NADPH oxidase-derived ROS influence NET formation inside the cell appear complex, and are still not fully conclusive. There is accumulating evidence that NADPH oxidase-derived ROS acting at the intracellular level are capable, and in some cases requisite, to initiate the formation of NETs. Experiments with ^1^O_2_ scavengers have confirmed singlet oxygen involvement in NADPH oxidase-dependent NET formation in human neutrophils upon stimulation with PMA [74]. Experimental inhibition [32,84] and mutation-caused failure [85] of NADPH oxidase has been shown to prevent NET formation. NADPH oxidase-deficient neutrophils of mutant mice and of humans with chronic granulomatous disease (CGD) are not able to form NETs [18,32,86]. However, although recent work has indicated that it is intracellular ROS levels that direct signaling in favor of autophagy/NETosis (see Section 2 above), the details of how this occurs are still not fully understood. Particularly it remains unclear how ROS influence the main features of NETosis, *i.e*., chromatin decondensation, histone citrullination, binding of enzymes to DNA, and membrane rupture. Present research has only provided pieces of a much larger puzzle. There is evidence indicating that in addition to their role in NE activation, ROS enable the release of NE and MPO from the azurophilic granules in the neutrophil cytoplasm. This is a prerequisite for the translocation of these enzymes to the nucleus, where NE aids histone degradation and MPO chromatin relaxation [13,59,87,88]. Additionally, ROS act to facilitate the citrullination of histone proteins by peptidyl arginine deiminase type 4 (PAD4) [89]. All these together are thought to promote nucleosome disassembly, chromatin decondensation, and the rupture of intracellular membranes. An upstream requirement of NADPH oxidase-dependent NET formation, and specifically also of PAD4-mediated histone citrullination, seems to be a rise in cytosolic calcium concentration via influx from the ER and/or the extracellular space [37,90].

The work of Parker *et al.* [91] indicates that the involvement of NADPH oxidase-derived ROS in the regulatory pathways of NET formation, just as the pathways themselves, vary depending upon the inducing molecular stimuli. These authors found that NETosis requires NADPH oxidase-derived ROS when induced with PMA or by bacterial stimulation, but not if the induction occurs via calcium influx mediated by the bacterial calcium ionophore ionomycin (see Section 8 below). Other work confirmed the role of NADPH oxidase-derived ROS in PMA-induced NET release from human neutrophils and demonstrated involvement of the MAPK/ERK pathway [84]. Similarly, MPO was found essential only after PMA induction, while bacteria induced NETs were formed without it [91]. Probably in partial contrast to this, Metzler *et al.* [92] found that functional MPO is a strict requirement for NET formation when comparing the NET-forming ability of neutrophils from MPO-deficient subjects and healthy donors after stimulation with PMA and opsonized *Candida albicans* cells. Work investigating NET formation in neutrophilic granulocytes of carp suggests that a stimulus-dependent selective requirement of ROS is an evolutionarily conserved pattern of vertebrate phagocytes [8].

## 4. NADPH Oxidase-Dependent NETosis Is a Matter of Delicate Coordination, Depending on Various Co-Factors

There are several lines of evidence to indicate that the regulatory influences behind the NADPH oxidase-dependent pathway of autophagy/NETosis induction are far from straightforward, and much is still incompletely understood. Perhaps the most fundamental question in this respect is under what circumstances NADPH oxidase-derived ROS signaling promotes autophagy/NETosis rather than cell death through apoptosis? This is because ROS such as H_2_O_2_ have also been found to promote neutrophil apoptosis, e.g., by caspase activation via the sphingolipid ceramide or the lysosomal aspartyl protease cathepsin D [93,94,95]. Such dual response is evident from recent work on the mouse lung showing that *Aspergillus* infection in the presence of functional NADPH oxidase enables NETosis while at the same time promoting neutrophil apoptosis [18]. Present knowledge of factors that affect whether NADPH oxidase-derived ROS direct a neutrophil into the autophagy/NETosis pathway is limited. Particular focus may be placed on the following aspects (see also Figure 2):

▪ **Cathepsin C**. Recent work investigating NET formation in patients with Papillon-Lefèvre syndrome suggests that the cycteine protease cathepsin C interferes with the interplay between NADPH oxidase-derived ROS and NETosis (albeit without entailing a substantial deficit in general immune defense) [83].
▪ **DOCK proteins**. A co-regulatory role in NADPH oxidase-dependent NETosis has been established for “dedicator of cytokinesis” (DOCK) proteins. Via their function as activators of Rac GTPases, DOCK proteins are involved in both neutrophil chemotaxis [96] and NADPH oxidase-dependent ROS production, the latter entailing a massive reduction in NET-forming ability in DOCK2^−/−^ individuals and to an almost complete loss of this ability in DOCK2^−/−^/DOCK5^−/−^ double deficient individuals in the murine test system [97].
▪ **Zinc**. Intracellular zinc ion (Zn^2+^) concentration has been identified as a co-regulator in PMA-induced protein kinase C mediated NET formation, which depends on the NADPH oxidase-derived ROS [98].
▪ **mTOR-related pathways**. Data from *in vitro* experimentation with human neutrophils using fMLP as inducing agent suggest that a central role in the ROS-mediated regulation toward NETosis is played by pathways involving the mTOR serine/threonine kinase [99]. Specifically the (PI3K/AKT)/mTOR pathway has been confirmed as a ROS sensitive negative regulator of autophagy [57,100,101], and is also gaining attention in relation to autophagy-related NETosis [44,59]. Moreover, also in this case, commitment to autophagy/NETosis was not found to be mandatory, as the ROS mediated impairment of mTOR activity may also terminate in apoptosis [57,102,103], so that additional factors must be assumed to play a role. Relevant regulatory influence in this respect will result from whether the molecular inducers activate NETosis in an mTOR-dependent manner, or along other pathways, as shown for IL-8 [26,27,90].
▪ **Protein kinase C**. The observations of Neeli and Radic [104] suggest that the calcium-dependent regulation of histone deimination by PAD4 (see also Section 3 above) is influenced by an intricate antagonism between the alpha and zeta isoforms of protein kinase C (PKC).
▪ **Extracellular matrix**. Important co-factors seem to be located in the extracellular matrix. Recent work on human neutrophil responses to fungal (*Candida albicans*) infection indicates that ubiquitous matrix constituents such as fibronectin may be attached with a significant role in deciding between respiratory burst behavior and NETosis [105]. This work demonstrates that neutrophils, when exposed solely to *Candida*-derived beta-glucan, activate ROS production but not NETosis. Simultaneous presence of fibronectin and beta-glucan, by contrast, leads to the suppression of ROS production and to rapid NET generation. The type of NETosis found depends on MAPK/ERK but not on ROS, and exhibits fine structural features that are strikingly similar to those shown for ROS independent *Staphylococcus* induced NETosis by Pilsczek *et al.* [31] (see also Section 7 below). This illustrates that extracellular matrix components may exert a more complex influence on the co-regulation of ROS production and NETosis than presently assumed. These results are in close agreement with experimental findings prior to the detection of (N)ETosis. Testing neutrophils adhering to substrates including collagen IV, laminin, thrombospondin, heparan sulfate proteoglycan (HSP) and again fibronectin during respiratory bursts primed by PMA and TNF-alpha, Borgquist *et al.* [106] showed a highly variable ROS production depending upon the extracellular matrix present. Recent work demonstrates that a major role in extracellular matrix effects on ROS-dependent NETosis mediated by NADPH oxidase and MPO is likely to be played by immobilized immune complexes that bind to neutrophil FcγRIIIb receptors [29].
▪ **Microbial-derived substances**. A further source of co-factors influencing the regulation of ROS-dependent NETosis is microbial-derived substances. This is demonstrated by recent work showing that gram-positive bacteria derived peptide bacteriocins containing polycyclic thioether amino acids (so-called lantibiotics, such as nisin) enhance levels of NADPH oxidase-derived intracellular ROS and induce NETosis in human neutrophils in a dose-dependent manner [107].

All this strengthens the view that ROS-dependent NETosis in neutrophils is modulated in a complex manner by integration of multiple stimuli (see also Byrd *et al.* [105]). However, in the light of the results of a recent investigation of NET-forming ability in cathepsin C-deficient individuals (see point on cathepsin above), it may be necessary to add that failure of ROS-mediated NET formation need not entail a substantial deficit in general immune defense [83].

## 5. Subtypes of NADPH Oxidase and Extracellular ROS

In addition to the present incomplete knowledge of co-factors, understanding of the role of ROS in the regulation of subcellular events during NETosis is further complicated by a variety of other parameters, such as the different pathways of NADPH oxidase activation depending on the type of external inducers. These uncertainties are exemplified by the observation that it is still not clear whether neutrophil NADPH oxidase may be divided into two subtypes that are distinct from each other by both their location and the involvement of PI3K in their activation pathways. This divergence has been demonstrated by inhibition experiments with the PI3K inhibitor wortmannin (a fungal steroid metabolite), which reliably blocks neutrophil NADPH oxidase activation if depending on PI3K pathways. It was first shown for activation induced by *N*-formylmethionyl-leucylphenylalanine (fMLP), while PMA-induced activation was found to be resistant to inhibition by wortmannin [108]. The extended experiments of Karlsson *et al.* [71] then demonstrated that PMA is also able to induce a wortmannin-sensitive (*i.e*., PI3K-dependent) NADPH oxidase activation in neutrophils, which, however, resulted in only intracellular but not extracellular release of superoxide. Neutrophils were therefore thought to harbour two NADPH oxidase subtypes, one residing in the plasma membrane, the other in the membranes of the so-called specific granules within the neutrophil cyptoplasm. Activation of both subtypes was found to depend on MAPK/ERK kinase and protein phosphatase 1 and/or 2A, while diverging in dependence on PI3K, which was found essential only for the intracellular variant of the enzyme [71]. Such a role of the specific granules in NADPH oxidase-dependent intracellular ROS generation has been further corroborated by Ambruso *et al.* [109], while more recent work indicates that endosomes commonly assigned to the secretory vesicles are also involved [110]. In view of the emerging importance of ROS dependent PI3K-mTOR pathways in the control of NET formation via regulation of autophagy (e.g., [99,103], see also Section 2 above), this is a relevant point of uncertainty that would require further examination.

However, despite such uncertainties, other capacious evidence summarized by Bedard and Krause [40] suggests that all phagocyte/neutrophil NADPH oxidase contains NOX2/gp91^phox^ as central functional component, irrespective of localization. The model of NADPH oxidase function presented by these authors does not discriminate between plasma membrane- and granule membrane-localized subtypes of the enzyme. Instead, it depicts a flexible situation in which most NOX2 is, together with p22^phox^, localized in the membranes of intracellular granules as long as the cells maintain a resting state. According to this model, the granules fuse with the plasma membrane only after completion of subunit assembly and activation, thus enabling ROS release to the extracellular compartment. In addition, the model implicates that granule membrane-bound NOX2 may become functional as part of NADPH oxidase intracellularly, without the need for fusion with the surface membrane. This may be requisite for the proposed roles of ROS in signaling cascades for NETosis induction (see also [111]).

Irrespective of whether or not neutrophil ROS release to the extracellular and intracellular compartment is caused by distinct subtypes of NADPH oxidase, it is unclear how extracellular ROS contributes to NETosis induction. As reported above, most work on the role of ROS in NETosis signaling refers, as a matter of course, to intracellular ROS. However, knowledge is incomplete as to the extent to which these ROS originate from outside the cells. While it seems clear that there is an inward passage of superoxide anions ^●^O_2_^−^ and H_2_O_2_ through anion (Cl^−^) and aquaporin channels, respectively [112], it is not known how this transmembrane flux is balanced with ROS generation by intracellular NADPH oxidase (or other sources such as mitochondria). Some progress in this context has been made by the *in vitro* analyses of Kirchner *et al.* [41]. Testing the effects of various inhibitors of ROS generating enzymes (NADPH oxidase, SOD, MPO) and mitochondrial electron transport on NETosis in human neutrophils, these authors found that NADPH oxidase- and MPO-derived ROS, but not those from SOD and mitochondria, are important for NET release. This further underpins the potential importance of NADPH oxidase-generated superoxide, and perhaps also of spontaneously formed (not SOD-catalyzed) H_2_O_2_, in NETosis induction. However, as the NADPH oxidase inhibitor diphenyleneiodonium chloride (DPI) used in this work is likely to act similarly on plasma membrane-bound and intracellular NADPH oxidase [113,114], and, more importantly, also on mitochondrial OXPHOS flavoenzymes [115], the questions as to true sources of NETosis inducing ROS remain largely unresolved.

## 6. The Role of ROS from Sources other than NADPH Oxidase

Although much of the present knowledge on the role of ROS in the molecular signaling of ETosis/NETosis has been obtained from NADPH oxidase-dependent pathways, there are several lines of evidence suggesting that ROS contributing to the formation of NETs need not necessarily derive from pathways involving NADPH oxidase. It becomes increasingly evident that there are likely two distinct (main) types of ROS-dependent NETosis: one that requires ROS supply from NADPH oxidase and responds to NADPH oxidase blocking, and another that does neither of these, instead relying on other ROS sources (*cf.* [116]).

Substantial new information has come from work supplying singlet oxygen (^1^O_2_) to neutrophils from CGD patients and healthy humans via application of the photosensitizing agent *Photofrin* (porfimer sodium). Results indicate that singlet oxygen is likely to be able to induce NET formation independent from NADPH oxidase activation [74].

An alternative important source of ROS in eukarotic cells is mitochondrial oxidative phosphorylation (OXPHOS) complexes. Mitochondria are generally thought to be the largest contributors to intracellular ROS production (e.g., [39]). Work on oxidative stress levels has indicated that this might also apply to neutrophils [117], despite the fact that mature neutrophils are characterized by low mitochondrial content and reduced levels of oxidative phosphorylation [118]. Cytochrome C from mitochondrial OXPHOS complexes has been shown to support caspase activation for neutrophil apoptosis, the alternative cell fate to autophagy/NETosis [95]. ROS are relevant byproducts of mitochondrial energy supply to respiratory/oxidative bursts [40]. Intracellular ROS may be influenced by the dependence of mitochondrial OXPHOS complexes on NADPH-derived NADH as electron donors, entailing competition for NADPH between mitochondria and NADPH oxidase (e.g., [117]). This competition could be even stronger as NADPH oxidase may be able to utilize NADH as a second electron source in addition to NADPH, even though NOX2-containing phagocyte NADPH oxidases have a preference for the latter [40]. Despite a result to the contrary reported by Kirchner *et al*. [41], mitochondrial ROS production may thus, albeit in limited form, exert influence on intracellular ROS levels of ET-forming cells, even in neutrophils. This conclusion is in strong agreement with most recent evidence of a NADPH oxidase-independent pathway of NETosis that depends on mitochondrial ROS and is mediated by small conductance calcium-activated potassium channel 3 (SK3) (Figure 2). In contrast to NADPH oxidase-dependent NETosis, this pathway does not essentially require MAPK/ERK activation [116]. Further research will be required to determine the full scope of influence by mitochondrial ROS on the regulation of ROS-dependent NETosis, especially in the context of the close correlation with autophagy that is now apparent (see Section 2 above).

Another source of ROS not directly related to NADPH oxidase that is likely to play a particular role in ETosis-mediated inflammation is MPO. ETosis is certainly a key mechanism of MPO release to the extracellular compartment. The catalytic activity of MPO is partitioned between a halogenation cycle and a peroxidase cycle [119]. It is well known that MPO released by neutrophils during respiratory bursts catalyzes the oxidation of chloride (Cl^−^) bromide (Br^−^) and thiocyanate (SCN^−^) by H_2_O_2_ to hypochlorous acid (HOCl), hypobromous acid (HOBr) and hypothiocyanous acid (HOSCN), respectively. Although MPO itself may become inactivated by H_2_O_2_ [120], these oxidants support the phagocytes’ ability to kill pathogens. Both are, directly and via their secondary reactions, able to harm host tissues in various manners, leading to growth arrest, apoptosis or necrosis in a dose dependent manner [73,75,121,122]. Direct molecular influences include inactivation of thiol enzymes (specifically by HOSCN), modification of lipoproteins and perturbation of phosphorylation-dependent signaling pathways such as MAPK/ERK [119,120,121,123]. The potential feedback effects on the role of MAPK/ERK pathways in the induction of ET formation (see Section 2 and Section 3) are as yet largely undetermined. It will be a challenge to integrate the finding that extracellular products of MPO are unable to rescue ET formation in MPO-deficient neutrophils [92] into this context.

HOCl and HOBr contribute to the formation of ROS and radicals also via various secondary reactions. Both react, for example, with H_2_O_2_ to form singlet oxygen ^1^O_2_ and peroxyl radicals [76,124,125] (see also Section 3 above). Both also target thiols, thioethers, disulfides, amines and amides, leading to the formation of advanced oxidation products (AOPPs) that interfere with the structure and physiology of cells [119]. AOPP formation with the involvement of extracellular MPO is also likely to include Fenton reactions. They generate hydroxyl radicals (OH) from H_2_O_2_ catalyzed by transition metals (mainly iron) [39,40], accounting for the peroxidation of lipids with unsaturated fatty acyl residues, a long known factor in ROS-induced tissue damage [126,127,128]. At sites of inflammation, the extracellular iron required to allow for this is likely to be abundantly available from ferritin secretion by macrophages [129,130,131,132]. Further radicals derive from the peroxidase cycle of MPO, which performs one electron oxidation of a multitude of organic and inorganic substrates. These include amino acids (tyrosine, tryptophan), thiols, ascorbate, steroid hormones and urate, but also singlet oxygen ^1^O_2_ and nitric oxide NO [119].

Thus, MPO is not only an important constituent in the intracellular regulation of ET formation (see Section 3) but once extruded, in all probability also a key contributor to extracellular (interstitial) ROS accumulation and phagocyte-mediated tissue injury.

## 7. The Role of ROS in Non-Cell Death ETosis

In addition to the accumulating information on the characteristics of “standard type” ETosis, research over the last decade has also rendered evidence of alternative forms of ET formation. These forms deviate from the “standard type” both mechanistically and in that they may leave the donor cells viable, thus being referred to as non-cell death ETosis. But all these alternative forms have some relationship to ROS.

The earliest example of non-cell death ETosis is provided by Yousefi *et al.* [133]. This work describes a fast form of ET extrusion by eosinophils, which diverges from the “standard type” by its eruptive (“catapult-like”) nature, and also that it utilizes mitochondrial DNA thus avoiding instant cell death. The release of these ETs can be stimulated by IL-5 and LPS and proved sensitive to blocking with diphenyleneiodonium (DPI). Thus, it is likely that the underlying signaling is mediated by membrane bound receptors and depends on ROS. However, the exact circumstances of how this occurs have not been elucidated and may be complex. LPS alone is thought to be unable to activate NADPH oxidase while facilitating activation by subsequent triggers [2,66]. DPI has been shown to be not only an effective blocker of NADPH oxidase but also of other NAD(P)-dependent enzymes (such as glucose 6-phosphate dehydrogenase) and mitochondrial OXPHOS flavoenzymes [115]. It is thus unclear from where the ROS involved in this process derive and how they act. Similarly, it is not clear from both the morphological and the chemical points of view, how the mitochondrial DNA is combined with granule proteins to form these ETs, and how the lack of histones in mitochondrial DNA influences their function.

A second variant of ETosis that leaves the donor cells viable and relies, in all probability, on mitochondrial DNA has been established for human neutrophils in response to treatment with granulocyte/macrophage colony-stimulating factor (GM-CSF) and subsequent TLR4 or complement factor 5a (C5a) receptor stimulation. Also in this case, treatment with DPI leads to a complete block of (N)ET release, and neutrophils from ROS-deficient CGD patients failed to generate such type of ETs [30]. This indicates that the process depends on ROS, but with the same uncertainties/caveats regarding the use of the blocking agent DPI as mentioned for eosinophil ETosis above.

A further type of fast ETosis/NETosis occurring without instant cell death was recently described for human neutrophils in response to stimulation with *Staphylococcus aureus* bacteria [31]. Similar to eosinophil ETosis [133], this type is rather fast, with NETs being observable as early as 5–10 min after onset of stimulation. In contrast to both eosinophil ETosis [133] and the type of NETosis described by Yousefi *et al.* [30], the process is not based on the release of mitochondrial DNA. Instead, DNA from nuclear chromatin is extruded via vesicles that bud from the nuclear membranes and rupture after release into the extracellular environment. However, this third variant of ETosis seems to only represent the initial phase of a longer lasting cycle, terminating in “conventional” cell death NETosis. But even if so, it is remarkable that this initial phase appears to be entirely devoid of ROS regulation [31].

## 8. ROS-Independent ETosis

Evidence as to whether there are pathways of NETosis that are entirely independent of ROS appears incomplete and conflicting. Some uncertainty still remains as to the role of ROS from NADPH oxidase or other sources in the induction of NET formation by elevation of cytosolic calcium. Experiments testing the effects of bacterial ionomycin in human promyelocytic leukemia (HL-60) cells and neutrophils strongly suggested that calcium influx-mediated induction of NETosis utilizes a ROS-independent pathway [37,91]. In contrast to this, recent work also investigating calcium ionophore-mediated NETosis, provides evidence for a calcium activated pathway of NETosis that depends on mitochondrial ROS, while being independent of NADPH oxidase-derived ROS [116]. It remains to be clarified how these findings can be integrated.

An ambiguous but inspiring situation has developed regarding the relationship between uric acid (UA) and NETosis depending on NADPH oxidase-derived ROS. UA, an abundant terminal product of vertebrate nitrogen metabolism, can act as an antioxidant but also as a pro-oxidant and pro-inflammatory factor, depending on the particular conditions [134]. This dual nature is clearly reflected in the present literature on the role of UA in mammalian NETosis. Some recent work suggests that UA in the form of monosodium urate (MSU) crystals is a strong inducer of ROS-dependent NETosis, which can be inhibited by anti-oxidants such as butylated hydroxytoluene (BHT), butylated hydroxyanisole (BHA) and ascorbic acid [135]. However, other work testing the effects of non-crystalline UA in solution found a dose-dependent ambivalent influence [136]. Low concentrations of UA (1 mg/100 mL) exerted an inhibitory effect on NADPH oxidase-dependent NET formation, most likely due to the antioxidant potential of UA. High concentrations (8 mg/100 mL), were, by contrast, found to be potent inducers of NETosis. Tests with ROS-inhibited control neutrophils and neutrophils from ROS-deficient CGD patients demonstrated that NETosis induction by high UA levels occurs in a NADPH oxidase/ROS-independent manner, with nuclear factor “kappa-light-chain-enhancer” of activated B-cells (NF-κB) playing a role in the signaling pathway [136]. This is consistent with findings that NF-κB protein accumulates in the nuclei of PMA- or TNFα-stimulated neutrophils [137], and that reduced phosphorylation of the NF-κB p65 subunit by different inhibitors (ASA, BAY-11-7082, and Ro 106-9920) abrogates the formation of NETs [138].

## 9. Conclusions

There is rapidly growing evidence that ROS are able to interact with the formation of ETs in a multidimensional manner (Figure 2). This occurs either directly via the signaling cascades that allow for ET formation and release, or indirectly via influence on other factors that modulate the process. The interaction with ROS is likely to be an important determinant in the regulatory network that determines whether ETosis is beneficial or noxious. The ROS-ETosis interaction will need to be taken into account to understand the characteristics of virtually all kinds of inflammatory disease, and to improve their treatment.
